# Ultrasound-engineered chitosan nanocarriers improve the anticancer activity of dandelion phytochemicals in hepatocellular carcinoma cells

**DOI:** 10.3389/fphar.2026.1871616

**Published:** 2026-07-23

**Authors:** Ammar A. Bahauddin, Sameh A. Ahmed, Rawan Bafail, Samar F. Miski, Hashem K. Alghazzi, Eyad S. Alahmadi, Mazen A. Almuzaini, Adel Y. Abdulaal

**Affiliations:** 1 Department of Pharmacology and Toxicology, College of Pharmacy, Taibah University, Al-Madinah Al-Munawwarah, Saudi Arabia; 2 Department of Pharmacognosy and Pharmaceutical Chemistry, College of Pharmacy, Taibah University, Al-Madinah Al-Munawwarah, Saudi Arabia; 3 Department of Pharmaceutics and Pharmaceutical Industries, College of Pharmacy, Taibah University, Al-Madinah Al-Munawwarah, Saudi Arabia

**Keywords:** apoptosis, ionic gelation, phytochemical delivery, sustained release, taraxasterol

## Abstract

**Introduction:**

HCC remains a leading cause of cancer-related mortality, with limited effective and safe treatment options. Although dandelion (*Taraxacum officinale*)-derived phytochemicals show anticancer potential, their clinical use is restricted by poor solubility, instability, and low intracellular bioavailability. Ultrasound-engineered chitosan nanocarriers represent a promising strategy to overcome these limitations and enhance intracellular delivery. Accordingly, this study aimed to develop an ultrasound-assisted chitosan nanoparticle formulation encapsulating dandelion extract (UA-ChNPs/DE) to improve therapeutic efficacy against HCC cells.

**Methods:**

UA-ChNPs/DE nanoparticles were synthesized using an ultrasound-assisted ionic gelation method. Physicochemical properties were characterized using dynamic light scattering, SEM, and XRD. The *in vitro* release behavior was assessed over 72 h. Cytotoxic activity was evaluated in hepatocellular carcinoma cell lines (HepG2 and Huh7) and normal hepatic (LO2) cells using a cell viability assay, while apoptosis induction in HepG2 cells was analyzed by flow cytometry.

**Results:**

The optimized UA-ChNPs/DE exhibited a nanoscale particle size (∼105 nm), a narrow size distribution (PDI = 0.268), and a positive surface charge (+28.4 mV), indicating colloidal stability. Structural analysis confirmed successful encapsulation and partial amorphization of the phytochemical constituents. The nanoformulation demonstrated sustained release over 72 h with a reduced initial burst release compared with the free extract. Biological evaluation revealed significantly enhanced antiproliferative activity, with UA-ChNPs/DE achieving lower IC_50_ values in HepG2 (17.8 μg/mL) and Huh7 (21.5 μg/mL) cells compared with the free extract (49.4 and 54.8 μg/mL, respectively). In normal LO2 cells, reduced cytotoxicity (IC_50_ = 52.4 μg/mL) and higher selectivity index values (2.94 and 2.54) indicated preferential targeting of cancer cells. Additionally, UA-ChNPs/DE significantly increased apoptotic cell populations (∼40%), confirming enhanced apoptosis-mediated anticancer activity.

**Conclusion:**

UA-ChNPs effectively overcome key physicochemical and pharmacokinetic limitations of dandelion-derived compounds, resulting in improved anticancer efficacy, sustained-release behavior, and preferential cytotoxicity toward hepatocellular carcinoma cells. This nanoformulation represents a promising strategy for advancing plant-derived therapeutics in HCC.

## 1 Introduction

Hepatocellular carcinoma (HCC) is a pervasive and urgent global health issue, accounting for an estimated 85%–90% of all primary liver cancers around the world. HCC is one of the most common malignancies worldwide and a leading cause of cancer-related mortality, particularly in patients with cirrhosis (Mauro et al., 2025). Currently, available therapeutic approaches for HCC include surgical interventions, chemotherapeutics, and ionizing radiation (Krupa et al., 2024). However, these treatment modalities are often limited by suboptimal efficacy, systemic toxicity, and poor tumor selectivity. Although substantial progress has been achieved in cancer therapy, HCC still has a discouraging 5-year survival rate of approximately 25% (Kumari et al., 2018). Moreover, the overall survival rate remains around 20% within 5 years of diagnosis, highlighting the urgent need for safer and more effective therapeutic strategies (Dalzell et al., 2023).

Recently, herbal extracts have attracted considerable attention among researchers as alternative anticancer agents. These herbal extracts consist of complex mixtures of a wide variety of phytochemicals, many of which exhibit numerous pharmacological activities, such as antimicrobial, anticancer, anti-inflammatory, and antioxidant effects (Situmorang et al., 2024). This multi-targeted activity provides a promising therapeutic advantage by modulating multiple signaling pathways involved in tumor progression (Cui et al., 2025).

Dandelion (*Taraxacum officinale*), an herb used in traditional medicine for the treatment of various diseases, has received considerable attention for its pharmacological and anticancer activities (Wang et al., 2025). In various studies, the ethanolic extract of *Taraxacum officinale* exhibited inhibitory activity against various types of cancer, including hepatocellular carcinoma. One of the major bioactive compounds identified in *T. officinale* is taraxasterol (TS), which exhibits diverse pharmacological activities. Recent findings highlight the remarkable ability of TS to inhibit HCC cell proliferation, induce apoptosis, and promote cell-cycle arrest (Herrera Vielma et al., 2025). Furthermore, *in vivo* studies demonstrate the inhibitory role of TS in suppressing HCC tumorigenesis and reducing the expression of the proliferation marker Ki-67 in HCC-bearing mice. Most significantly, TS has been reported to enhance antitumor immunity by increasing CD4+ T-cell levels and facilitating their infiltration into HCC tissues. It acts through the suppression of anti-apoptotic proteins and the STAT3/IL-6 pathway to enhance antitumor immunity and suppress HCC progression (Ren et al., 2022).

Despite growing evidence supporting the anticancer properties of dandelion-derived compounds, their low solubility, instability, and poor bioavailability have substantially hindered their translational and therapeutic applicability (Yan et al., 2024). These limitations significantly restrict their intracellular uptake and therapeutic efficacy. Therefore, the development of efficient delivery systems is essential to overcome these challenges. Nanotechnology-based drug delivery systems have emerged as a powerful approach to enhance the therapeutic performance of natural compounds by improving their stability, cellular uptake, and controlled release behavior (Ghazal et al., 2024). Encapsulation of dandelion extract within nanoparticle systems can protect bioactive constituents from degradation, enhance bioavailability, and improve anticancer efficacy (Rîmbu et al., 2025).

Chitosan is a polysaccharide that is widely used as a nanoparticle carrier due to its biocompatibility, biodegradability, low toxicity, and excellent drug-delivery capabilities. Chitosan nanoparticles (ChNPs) have been shown to retain the biological activities of encapsulated substances while improving their bioavailability and anticancer activity (Stefanache et al., 2025). Moreover, the intrinsic cationic nature of chitosan facilitates interactions with negatively charged cellular membranes, thereby improving cellular uptake and intracellular delivery. Additionally, ChNPs have demonstrated inherent anticancer and antiangiogenic properties (Xu et al., 2009).

Ultrasound-assisted (UA) nanoparticle synthesis has emerged as an efficient and reproducible technique for the fabrication of chitosan-based nanocarriers. This approach enhances particle uniformity, improves loading efficiency, and optimizes physicochemical stability. Acoustic cavitation generated during ultrasound processing promotes efficient mixing, reduces aggregation, and enables the formation of uniformly dispersed nanoparticles without the need for harsh chemical conditions or elevated temperatures (Khoerunnisa et al., 2021). Additionally, UA techniques are particularly valuable for bioactive plant extracts since they allow efficient loading of phytochemical mixtures while maintaining structural and biological stability. Moreover, ultrasonic wave interactions with chitosan and plant extracts further improve surface homogeneity and facilitate sustained-release properties (Wu et al., 2022). Nevertheless, limited studies have systematically investigated the combined effect of ultrasound-assisted chitosan nanoencapsulation on enhancing the anticancer efficacy and selectivity of dandelion-derived compounds across multiple hepatocellular carcinoma models.

Despite the growing interest in nanotechnology-based delivery systems for phytochemicals, important knowledge gaps remain regarding the integration of ultrasound-assisted ionic gelation with chitosan nanoparticles to improve the stability, sustained release, selective cytotoxicity, and apoptosis-inducing activity of dandelion-derived bioactive compounds in hepatocellular carcinoma. Furthermore, comparative evaluation across multiple HCC cell lines and normal hepatic cells has been scarcely explored. Therefore, the aim of the present study was to develop UA-ChNPs/DE to improve the stability, delivery, and therapeutic efficacy of dandelion bioactive compounds. To the best of our knowledge, this is the first study to integrate ultrasound-assisted ionic gelation with chitosan nanoparticle engineering to enhance the anticancer efficacy and apoptosis-inducing activity of dandelion-derived phytochemicals in hepatocellular carcinoma. In addition, this study evaluates the antiproliferative activity, cytotoxic selectivity, and apoptosis-inducing potential of the nanoformulation across multiple HCC cell lines (HepG2 and Huh7) and normal hepatic cells (LO2). By integrating nanotechnology-based delivery systems with plant-derived therapeutics, this work provides a scientific foundation for the development of improved anticancer strategies targeting HCC.

## 2 Materials and methods

### 2.1 Materials

Chitosan (medium molecular weight, 50,000–190,000 Da, degree of deacetylation > 75%) and sodium tripolyphosphate (TPP) were purchased from Sigma-Aldrich (St. Louis, MO, United States). Glacial acetic acid (100%), sodium hydroxide, dimethyl sulfoxide (DMSO), Folin–Ciocalteu reagent, sodium carbonate, gallic acid standard, and all other analytical-grade reagents were also obtained from Sigma-Aldrich (St. Louis, MO, United States). Dandelion (*Taraxacum officinale*) dried plant material was obtained from an authenticated local supplier and processed in-house prior to extraction. Dulbecco’s Modified Eagle Medium (DMEM), fetal bovine serum (FBS), penicillin–streptomycin solution, and trypsin EDTA solution were purchased from Gibco (Thermo Fisher Scientific, United States). The MTT reagent and the Annexin V-FITC apoptosis detection kit with propidium iodide were procured from BioLegend Inc. (San Diego, CA, United States). Ultrapure distilled water was used throughout the experiments. HepG2 and Huh7 human hepatocellular carcinoma cell lines, along with LO2 normal hepatic cells, were obtained from King Abdullah University of Science and Technology (Saudi Arabia) and maintained under standard culture conditions. All chemicals and reagents were of analytical or cell-culture and used without further purification.

### 2.2 Dandelion extract preparation

Dandelion root extract was prepared by aqueous extraction followed by lyophilization, with minor modifications from a previously reported method (Shittu et al., 2025). Briefly, 50 g of fresh dandelion roots were thoroughly washed with distilled water to remove soil and surface impurities, air-dried, and mechanically macerated to obtain a homogenized plant mass. The macerated material was suspended in distilled water and stirred continuously at room temperature to facilitate extraction. The mixture was first filtered through a sterile nylon mesh to remove coarse fibrous materials. The filtrate was subsequently centrifuged at 5,000 × g for 6 min at ambient temperature and further clarified by vacuum filtration. The resulting supernatant was collected and filtered through a Büchner funnel followed by Whatman No. 1 filter paper to remove fine residual fibers and insoluble debris. The resulting extract was freeze-dried to obtain a dry powder with an extraction yield of 12.4% (w/w), calculated as the ratio of the weight of the lyophilized extract to the initial weight of dried plant material. For nanoparticle formulation and *in vitro* experiments, the dried extract was reconstituted to a defined stock concentration using sterile 1× phosphate-buffered saline (PBS). Working solutions were freshly prepared by dilution in cell culture medium immediately prior to application. The extract was standardized based on taraxasterol content using HPLC-DAD analysis to ensure batch-to-batch reproducibility.

### 2.3 Quantification of taraxasterol content of dandelion extract

Quantitative analysis of taraxasterol was performed according to a previously reported method (Sharma and Zafar, 2014) using a Shimadzu Prominence high-performance liquid chromatography (HPLC) system (Shimadzu, Japan) equipped with a diode array detector (SPD-M20A); detection range (190–1,100 nm) and controlled by LC-Solution software. The system comprised a quaternary gradient pump (LC-20AD), column oven (CTO-20A), autosampler (SIL-20AP), and communication bus module (CBM-20A). Chromatographic separation was achieved on a reverse-phase C18 column using an isocratic mobile phase consisting of methanol and acidified water (80:20, v/v). The flow rate was set at 0.8 mL/min, and the injection volume was 20 µL. Detection was conducted at a sensitivity of 0.1 AUFS over a wavelength range of 190–550 nm. The retention time of taraxasterol was approximately 2.5 min. A 10-min equilibration period was allowed between successive injections. Standard taraxasterol was dissolved in methanol to prepare calibration solutions at seven concentration levels (0.025–1.0 mg/mL). Each concentration level and sample solution were injected in triplicate. The calibration curve demonstrated excellent linearity (*r* ^2^ = 0.9989) over the tested concentration range.

### 2.4 Preparation of UA-ChNPs/DE

UA-ChNPs/DE were synthesized via ionic gelation combined with probe ultrasonication. Briefly, chitosan was dissolved in 1% (v/v) acetic acid to obtain a final concentration of 0.1% (w/v) and stirred overnight at room temperature until complete dissolution. The pH of the chitosan solution was adjusted to 4.5–5.5 using dilute sodium hydroxide solution. A freshly prepared aqueous solution of dandelion extract was added dropwise under continuous stirring to ensure homogeneous dispersion. The chitosan–extract mixture was then subjected to probe ultrasonication using a Vibra-Cell™ Ultrasonic Liquid Processor (Sonics & Materials, Inc., Newtown, CT, United States) operating at a fixed frequency of 24 kHz. Sonication was performed in pulsed mode (15 s ON/10 s OFF) at 40% amplitude for 10 min under ice cooling.

Following ultrasonic homogenization, ionic crosslinking was initiated by the dropwise addition of TPP solution, with concentrations evaluated in the range of 0.1%–1.0% (w/v). A TPP concentration of 0.2% (w/v) was selected as optimal based on nanoparticle size uniformity and colloidal stability. Nanoparticles were formed via electrostatic interaction between protonated chitosan and TPP. A brief secondary sonication step was applied to further refine particle size and improve dispersion uniformity. The nanoparticles were collected by centrifugation, washed, and either used immediately or lyophilized for further analysis. Blank chitosan nanoparticles (ChNPs) prepared using the same procedure, except that an equivalent volume of distilled water was substituted for the dandelion extract.

### 2.5 Ultrasonic setup for nanoparticle preparation

UA emulsification and nanoparticle formation were performed using a Vibra-Cell™ Ultrasonic Liquid Processor (Sonics & Materials, Inc., Newtown, CT, United States) operating at a fixed frequency of 24 kHz. The system consisted of a power generator, a converter, and a horn microtip (22 mm in diameter). The generator converted 60 Hz alternating electrical power into 24 kHz ultrasonic energy, while the converter transformed this energy into mechanical vibrations via an internal piezoelectric lead zirconate titanate (PZT) transducer. These vibrations were transmitted through the horn microtip immersed directly into the chitosan–extract mixture. The maximum output power of the system was 750 W; however, the operating amplitude was limited to 40% to prevent excessive cavitation intensity and thermal degradation. Sonication was conducted in pulsed mode (15 s ON followed by 10 s OFF) to enhance nanoparticle homogeneity and minimize overheating. An ice bath was used throughout the process to maintain temperature control and preserve formulation stability.

### 2.6 Physicochemical characterization

#### 2.6.1 Particle size distribution and zeta potential analysis

The mean particle size, polydispersity index (PDI), and zeta potential of UA-ChNPs/DE were determined using a Microtrac S3500 particle size analyzer (Microtrac Inc., Montgomeryville, PA, United States). Samples were diluted 10-fold with distilled water prior to obtaining an appropriate scattering intensity for DLS analysis. All measurements were performed in triplicate at 25 °C.

#### 2.6.2 Differential scanning calorimetry (DSC)

Thermal properties of pure chitosan nanoparticles, dandelion extract, and UA-ChNPs/DE were analyzed using a Netzsch DSC apparatus (Netzsch F3 Maia®, Selb, Germany). Samples were sealed in aluminum pans and heated over a temperature range of 25 °C–300 °C at a heating rate of 10 °C/min under a nitrogen atmosphere. Thermograms were analyzed to assess thermal transitions and potential interactions between the polymer matrix and the encapsulated extract.

#### 2.6.3 X-ray powder diffraction (XRPD) analysis

Crystalline properties of UA-ChNPs/DE and control samples were analyzed using a Shimadzu XRD-6000 diffractometer (Shimadzu Corporation, Kyoto, Japan) equipped with a copper anode. The instrument was operated at 40 kV and 30 mA using Cu Kα radiation. X-ray powder diffractograms were recorded over a 2θ range of 10°–60° at a scanning speed of 0.04°/min. Diffraction patterns were evaluated to determine changes in crystallinity following encapsulation.

#### 2.6.4 Scanning electron microscopy (SEM)

The surface morphology of chitosan nanoparticles and UA-ChNPs/DE was examined using scanning electron microscopy. Lyophilized samples were mounted on aluminum stubs, sputter-coated with gold, and observed under appropriate accelerating voltages to evaluate particle shape, surface morphology, and dispersion characteristics.

### 2.7 *In vitro* release kinetics and diffusion modeling of dandelion extract from UA-ChNPs/DE

The *in vitro* release of dandelion extract from UA-ChNPs/DE was evaluated using a dialysis bag diffusion method under sink conditions. Briefly, 5 mg of UA-ChNPs/DE, corresponding to 10.20 ± 0.34 μg/mL of taraxasterol, was dispersed in 3 mL of phosphate-buffered saline (PBS, pH 7.4). The dispersion was transferred into a pre-soaked dialysis membrane (molecular weight cut-off: 7,000 Da), which was tightly sealed and immersed in 50 mL of release medium. The system was maintained at 37 °C ± 0.5 °C under continuous gentle agitation at 50 rpm to simulate physiological conditions and ensure uniform diffusion. At predetermined time intervals, 1 mL aliquots of the release medium were withdrawn and immediately replaced with an equal volume of fresh, pre-warmed PBS to maintain sink conditions, a constant release volume, and an appropriate concentration gradient throughout the experiment. The released extract was quantified based on taraxasterol content using HPLC-DAD analysis. All experiments were performed in triplicate, and the results were expressed as mean ± standard deviation (SD). The cumulative percentage of dandelion extract released at each time point was calculated using the following Equation 1:

$$\text{The cumulative release }\left(\%\right)=\frac{\;\text{Amount}\;\text{of}\;\text{the}\;\text{extract}\;\text{released}\;\text{at}\;\text{each}\;\text{time}\;\text{point}\;\times \;100}{\text{Total}\;\text{amount}\;\text{of}\;\text{extract}\;\text{loaded}}$$ (1)

To further elucidate the release mechanism, the experimental results were fitted to kinetic models. The Higuchi model was applied to evaluate diffusion-controlled release from the polymeric matrix, as it is commonly used to describe diffusion-governed release from polymeric matrix systems (Petropoulos et al., 2012), according to the Equation 2:

$$Q_{t}=k_{H}\;t^{1/2}$$ (2)

where $Q_{t}$ is the cumulative amount of drug released at time t , and $k_{H}$ is the Higuchi dissolution constant.

### 2.8 Determination of encapsulation efficiency, loading capacity, and production yield

Encapsulation efficiency (EE%), drug loading capacity (DL%), and nanoparticle production yield were determined for the optimized UA-ChNPs/DE formulation. Following nanoparticle preparation, the dispersion was centrifuged at 15,000 rpm for 30 min to separate the nanoparticles from the aqueous phase. The supernatant containing unencapsulated taraxasterol was collected, appropriately diluted, and analyzed using the validated HPLC-DAD method described in Section 2.3. The amount of encapsulated taraxasterol was calculated as the difference between the total amount of taraxasterol initially used during formulation and the amount of free taraxasterol detected in the supernatant.

The encapsulation efficiency (EE%) was calculated according to Equation 3:

$$\text{EE }\left(\%\right)=\left[\left(\text{Total taraxasterol}-\text{Free taraxasterol}\right)/\text{Total taraxasterol}\right]\times 100$$ (3)

The loading capacity (DL%) was calculated according to Equation 4:

$$\text{DL }\left(\%\right)=\left[\left(\text{Total taraxasterol}-\text{Free taraxasterol}\right)/\text{Weight of recovered nanoparticles}\right]\times 100$$ (4)

The nanoparticle production yield was determined according to Equation 5:

$$\text{Production Yield }\left(\%\right)=\left(\text{Weight of recovered nanoparticles}/\text{Total weight of starting materials}\right)\times 100$$ (5)

### 2.9 Cell culture conditions

HepG2 and Huh7 human hepatocellular carcinoma cell lines, along with LO2 normal hepatic cells, were used to evaluate cytotoxicity and selectivity. All cell lines were cultured in Dulbecco’s Modified Eagle Medium (DMEM) supplemented with 10% (v/v) fetal bovine serum (FBS) and 1% (v/v) penicillin–streptomycin. Cells were maintained at 37 °C in a humidified atmosphere containing 5% CO_2_. Subculturing was performed using trypsin–EDTA upon reaching approximately 70%–80% confluence to maintain optimal growth conditions and cell viability. All cell-based experiments were performed as three independent experiments (N = 3), and each treatment condition was evaluated in triplicate wells within each experiment (n = 3).

### 2.10 *In Vitro* cytotoxicity assessment and selectivity index

The cytotoxic effects of DE, blank ChNPs, and UA-ChNPs/DE were evaluated using the MTT assay with minor modifications to the standard protocol (Mosmann, 1983). HepG2, Huh7, and LO2 cells were seeded in 96-well plates at a density of 1 × 10^4^ cells per well and incubated overnight. Cells were treated with increasing concentrations (1–50 μg/mL) for 24 h. Following treatment, MTT solution was added, and the cells were incubated for 4 h at 37 °C to allow formazan formation. The crystals were dissolved in DMSO, and absorbance was measured at 570 nm using a microplate reader. Cell viability was expressed as a percentage relative to untreated controls, and IC_50_ values were calculated using nonlinear regression analysis.

To evaluate the therapeutic selectivity of the nanoformulation, the selectivity index (SI) was calculated using the following Equation 6:

$$\text{SI}={\text{IC}}_{50}\;\left(\text{normal cells}\right)/{\text{IC}}_{50}\;\left(\text{cancer cells}\right)$$ (6)

An SI value greater than 2 was considered indicative of selective cytotoxicity toward cancer cells.

### 2.11 Apoptosis analysis by annexin V-FITC/PI staining

Apoptosis induction was quantitatively assessed using Annexin V-FITC/propidium iodide (PI) dual staining followed by flow cytometric analysis. HepG2 and Huh7 cells were treated with UA-ChNPs/DE at their respective IC_50_ concentrations for 24 h. Untreated cells served as negative controls. Following treatment, cells were harvested, washed twice with cold phosphate-buffered saline (PBS), and resuspended in binding buffer. Cells were then incubated with Annexin V-FITC and PI for 15 min at room temperature in the dark, according to the manufacturer’s protocol. Samples were immediately analyzed by flow cytometry to quantify the proportions of viable (Annexin V^−^/PI^−^), early apoptotic (Annexin V^+^/PI^−^), late apoptotic (Annexin V^+^/PI^+^), and necrotic (Annexin V^−^/PI^+^) cells. Data were expressed as percentages relative to the total cell population.

### 2.12 Statistical analysis

All experiments were performed in triplicate, and data are presented as mean ± standard deviation (SD) derived from three independent experiments (N = 3), each performed using triplicate measurements (n = 3). Statistical analyses were conducted using IBM SPSS software. Comparisons among treatment groups were carried out using one-way analysis of variance (ANOVA) followed by Tukey’s multiple-comparison *post hoc* test. Differences were considered statistically significant at *P* < 0.05.

## 3 Results

### 3.1 Optimization of ultrasound-assisted UA-ChNPs/DE preparation

The preparation of UA-ChNPs/DE was optimized by systematically evaluating key formulation and process parameters, including ultrasonic amplitude, power output, sonication time, extract incorporation strategy, and TPP concentration. The influence of these variables on particle size, polydispersity index (PDI), and zeta potential was assessed using dynamic light scattering (DLS). Ultrasonic amplitude was evaluated over a range of 20%–60% of the maximum instrument output (750 W). At low amplitudes (20%–30%), nanoparticles exhibited larger particle sizes and broader size distributions. Increasing the amplitude to 40% resulted in a marked reduction in particle size and improved size uniformity. Further increases beyond 40% resulted in increased particle size and PDI, indicating reduced dispersion stability due to excessive cavitation. Therefore, an ultrasonic amplitude of 40% was selected as the optimal condition. The ultrasonic power output was evaluated over a range of 100–750 W. At power levels below 200 W, incomplete nanoparticle formation was observed. A progressive reduction in particle size and improved surface charge stability were achieved with increasing power output, with the most uniform nanoparticles obtained at 750 W. No further improvement was observed beyond this level; therefore, 750 W was selected as the optimal power.

Sonication time was evaluated over the range of 2–20 min under both continuous and pulsed modes. Short sonication durations (<6 min) resulted in insufficient nanoparticle formation and broad size distributions. A sonication duration of 10 min produced nanoparticles with reduced particle size and stable zeta potential. Prolonged sonication (>15 min) led to increased particle size and PDI, likely due to re-agglomeration. Pulsed sonication (15 s ON/10 s OFF) consistently yielded smaller and more uniformly dispersed nanoparticles. Accordingly, 10 min of pulsed sonication was selected as the optimal condition.

The concentration of TPP used for ionic crosslinking was evaluated over a range of 0.1%–1.0% (w/v). At low TPP concentrations (0.1%), nanoparticle formation was incomplete, resulting in unstable dispersions. Increasing the TPP concentration to 0.2% led to the formation of well-defined nanoparticles with a narrow size distribution and stable surface charge. Further increases (>0.3%) resulted in increased particle size and PDI due to excessive crosslinking and aggregation. At higher concentrations (∼1.0%), pronounced aggregation and reduced colloidal stability were observed.

The incorporation of dandelion extract into the chitosan solution prior to crosslinking was evaluated. Post-formation addition of the extract resulted in increased particle size and higher PDI, indicating poor encapsulation efficiency. In contrast, adding dandelion extract to the chitosan solution before TPP-mediated crosslinking resulted in smaller particle sizes and narrower size distributions. This approach enabled improved dispersion of phytochemicals within the polymer matrix, leading to enhanced nanoparticle uniformity and stability.

Based on the optimization studies, the final preparation conditions selected for UA-ChNPs/DE nanoparticles were as follows: incorporation of dandelion extract into the chitosan solution prior to crosslinking, ultrasonic amplitude of 40%, power output of 750 W, 10 min of pulsed sonication (15 s ON/10 s OFF), and a TPP concentration of 0.2% (w/v). These conditions consistently produced nanoparticles with optimal size distribution, low polydispersity, and stable surface charge and were therefore used for subsequent characterization and biological evaluation.

### 3.2 Physicochemical characterization

#### 3.2.1 Particle size distribution and surface charge of UA-ChNPs/DE

**FIGURE 1:**
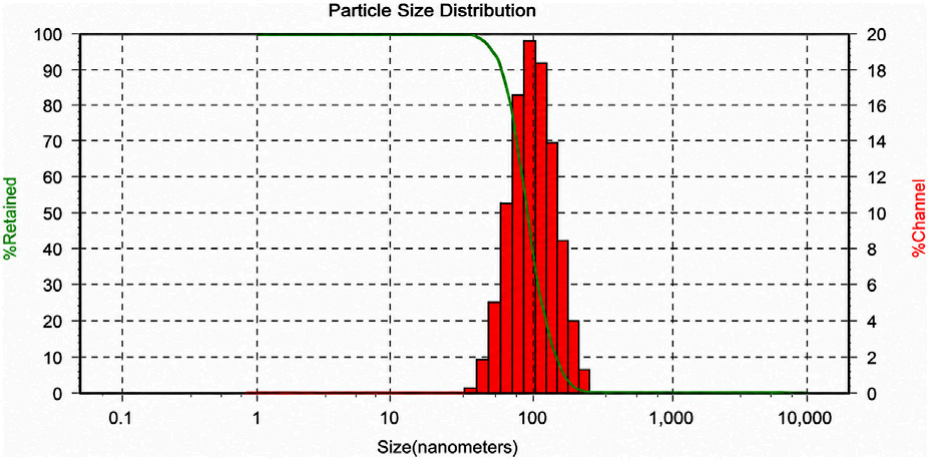
Particle size distribution of UA-ChNPs/DE prepared by the ultrasound-assisted technique at 40% amplitude, 750 W, for 10 min in pulsed mode.

The particle size distribution of UA-ChNPs/DE is shown in Figure 1. DLS analysis revealed that UA-ChNPs/DE exhibited an average particle size of 105.2 nm, confirming the successful formation of nanosized particles. The PDI was 0.268, reflecting a relatively narrow and uniform size distribution. The D10, D50, and D90 values (78.6, 104.3, and 138.7 nm, respectively) further confirmed a homogeneous particle population. Approximately 80% of the particles were distributed within the 79–139 nm range, indicating effective size control during ultrasonic processing.

Zeta potential measurements were performed to determine the surface charge of UA-ChNPs/DE. The nanoparticles exhibited a positive zeta potential of +28.4 ± 1.6 mV, reflecting the presence of protonated amino groups on the chitosan surface. This positive surface charge indicates good colloidal stability and the potential for enhanced cellular interactions. In comparison, free dandelion extract exhibited a markedly lower zeta potential of −6.3 ± 0.9 mV. This finding highlights the stabilizing effect of the chitosan nanocarrier system.

#### 3.2.2 Surface morphology analysis by scanning electron microscopy

**FIGURE 2:**
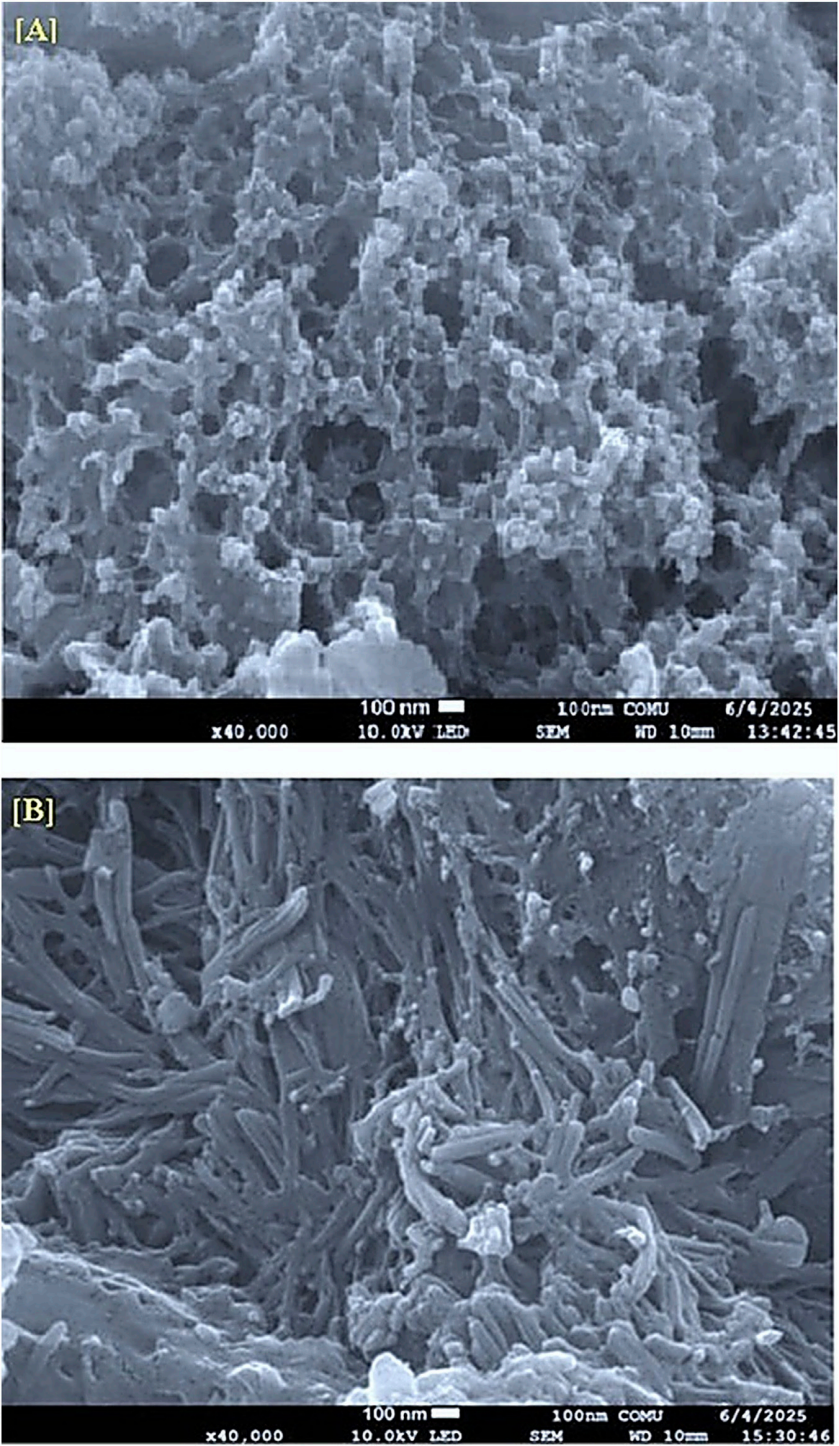
Surface morphology of blank UA-ChNPs **(A)** and UA-ChNPs/DE **(B)** as observed by scanning electron microscopy (SEM).

Scanning electron microscopy was employed to examine the surface morphology of blank chitosan nanoparticles and UA-ChNPs/DE, as shown in Figures 2A,B, respectively. Blank chitosan nanoparticles exhibited spherical morphology with smooth surfaces, characteristic of ionically crosslinked chitosan systems. In contrast, UA-ChNPs/DE maintained a spherical morphology with slightly roughened surfaces. This surface roughness may be attributed to the incorporation of phytochemical constituents within the polymer matrix. Importantly, the nanoparticles appeared discrete and well-separated, with no evidence of aggregation or structural collapse. These findings confirm that ultrasound-assisted processing preserved particle integrity and promoted uniform morphology.

#### 3.2.3 Crystalline properties assessed by X-Ray powder diffraction

**FIGURE 3:**
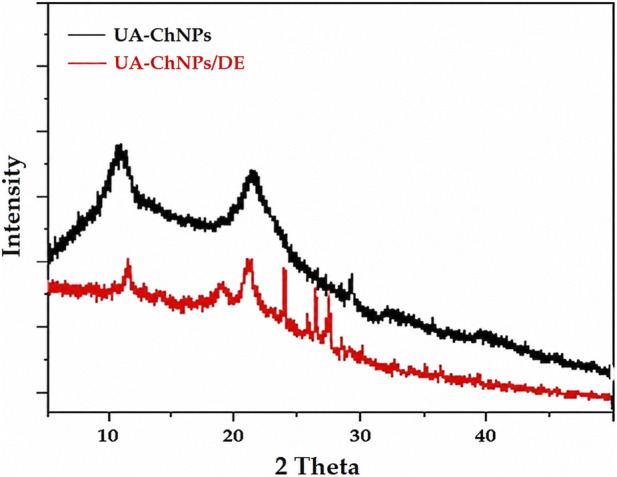
X-ray powder diffraction (XRPD) pattern of blank UA-ChNPs and UA-ChNPs/DE.

The crystalline characteristics of dandelion extract, blank chitosan nanoparticles, and UA-ChNPs/DE were evaluated using X-ray powder diffraction analysis (Figure 3). The pure extract exhibited distinct diffraction peaks, indicating partial crystallinity. Blank chitosan nanoparticles exhibited a broad diffraction halo, characteristic of an amorphous polymeric structure. Upon encapsulation, a marked reduction in, or complete disappearance of, the crystalline peaks were observed in UA-ChNPs/DE. This finding indicates successful incorporation of the extract and its transition toward an amorphous state. These findings suggest that ultrasound-assisted encapsulation effectively modified the solid-state properties of phytochemicals, which may enhance dissolution and bioavailability.

### 3.3 Controlled release behavior and diffusion kinetics of UA-ChNPs/DE

**FIGURE 4:**
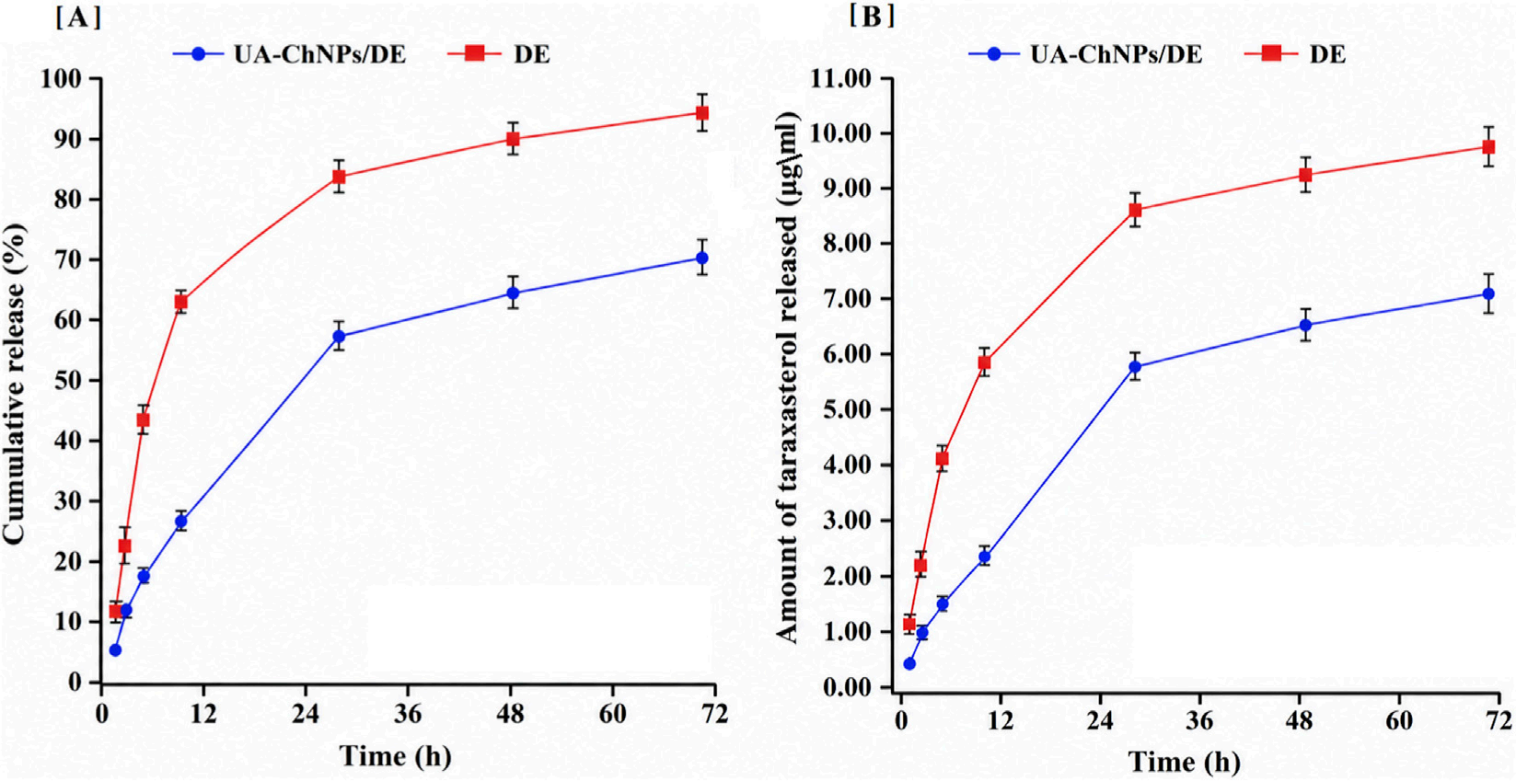
*In vitro* release profiles of taraxasterol from free dandelion extract and UA-ChNPs/DE in PBS (pH 7.4) at 37 °C using the dialysis bag diffusion method. **(A)** Cumulative percentage release of taraxasterol versus time. **(B)** Cumulative amount of taraxasterol released versus time. The free extract and nanoformulation were evaluated using equivalent taraxasterol concentrations. Data are presented as mean ± SD (n = 3).

The *in vitro* release profiles of free dandelion extract (DE) and UA-ChNPs/DE in PBS (pH 7.4) at 37 °C using the dialysis bag diffusion method are presented in Figure 4A. Release was quantified based on taraxasterol content using the validated HPLC-DAD method, and the corresponding cumulative amount of taraxasterol released over time is presented in Figure 4B. Free DE exhibited a rapid release pattern, with 23.6% ± 1.8% released within the first hour, increasing sharply to 63.4% ± 2.1% at 8 h. The cumulative release reached 86.2% ± 2.4% at 24 h and approached near-complete release (96.5% ± 1.7%) at 72 h, indicating the absence of sustained-release control in the free extract system. When expressed as the cumulative amount of taraxasterol released, free DE achieved 9.84 µg of the initial 10.20 µg taraxasterol content by 72 h.

In contrast, UA-ChNPs/DE demonstrated a markedly sustained-release profile. An initial release of 12.4% ± 1.5% was observed at 1 h, followed by a gradual increase to 28.0% ± 2.0% at 8 h. The cumulative release reached 59.3% ± 2.5% at 24 h and further increased to 70.6% ± 3.1% at 72 h. This corresponded to a cumulative taraxasterol release of approximately 7.20 μg at 72 h, confirming the ability of the chitosan nanoparticle system to prolong release relative to the free extract. This reduced initial burst and prolonged release behavior indicate effective encapsulation of the extract within the chitosan matrix and controlled diffusion of phytochemicals over time. Compared with free DE, the nanoformulation significantly modulated the release kinetics by limiting rapid diffusion and extending drug availability, which is advantageous for sustained therapeutic action. The cumulative-release profile shown in Figure 4B further illustrates the pronounced difference in taraxasterol release behavior between the free extract and nanoparticle formulations throughout the study period.

To further elucidate the release mechanism, the experimental data were fitted to the Higuchi model, which describes diffusion-controlled release from a polymeric matrix. The Higuchi model was selected because it is one of the most widely accepted models for evaluating diffusion-governed release from polymeric drug-delivery systems and is particularly applicable to matrix-based formulations in which the release process is controlled primarily by diffusion through a hydrated polymeric network (Petropoulos et al., 2012; Naskar et al., 2019). The Higuchi plot exhibited a strong linear relationship (*R* ^2^ > 0.98), suggesting that the release of dandelion extract from UA-ChNPs/DE is primarily governed by diffusion through the chitosan–TPP matrix. The Higuchi dissolution constant (K_H_ = 9.4%. h^−1/2^) indicates a controlled release behavior and will be refined upon final model fitting. In contrast, the free extract showed poor correlation with the Higuchi model due to its rapid and uncontrolled release behavior, confirming the absence of a diffusion-regulated mechanism. These findings demonstrate that UA-ChNPs effectively convert the release profile of dandelion extract from an immediate-release system into a sustained, diffusion-controlled delivery platform.

### 3.4 Encapsulation efficiency, loading capacity, and production yield of UA-ChNPs/DE

The pharmaceutical performance of the optimized UA-ChNPs/DE formulation was evaluated by determining its encapsulation efficiency (EE%), drug loading capacity (DL%), and nanoparticle production yield. The formulation exhibited an EE% of 20.5%, indicating successful incorporation of dandelion extract constituents within the chitosan nanoparticle matrix. The DL% was 0.21%, reflecting the amount of taraxasterol associated with the recovered nanoparticle mass. Furthermore, the nanoparticle production yield reached 60.0%, demonstrating satisfactory recovery of the formulation following the ultrasound-assisted ionic gelation process. These results confirm the successful fabrication of UA-ChNPs/DE and demonstrate the ability of the chitosan-based nanocarrier system to incorporate and retain dandelion phytochemicals. The observed encapsulation characteristics are consistent with the sustained-release behavior demonstrated in the *in vitro* release study, where the nanoparticle formulation exhibited a reduced initial burst release and a prolonged release profile compared with the free extract. These findings suggest that encapsulation within the chitosan matrix contributed to controlled phytochemical release and may have enhanced the biological performance of the formulation observed in subsequent cellular studies.

It should be noted that encapsulation efficiency was estimated based on the quantified taraxasterol content associated with the formulation and the experimentally measured taraxasterol content of the dandelion extract used during nanoparticle preparation. A complete mass-balance determination involving nanoparticle disruption and total-content recovery analysis was not performed. Future studies will incorporate comprehensive recovery assessments following nanoparticle dissolution to further validate the encapsulation efficiency estimates and account for potential formulation-related losses.

### 3.5 *In Vitro* cytotoxicity assessment and selectivity index

#### 3.5.1 Dose-dependent cytotoxicity in HepG2 cells and IC_50_ analysis

The cytotoxic activity of UA-ChNPs/DE, free DE, and blank ChNPs was evaluated in HepG2 cells using the MTT assay following 24 h of exposure. A clear concentration-dependent reduction in cell viability was observed (Table 1). At 1.0 μg/mL, no significant differences were observed among the treatment groups (*P* = 0.214), indicating minimal cytotoxicity at this concentration. However, significant differences emerged at concentrations ≥10 μg/mL (*P* < 0.001), where UA-ChNPs/DE reduced cell viability to 60.8% ± 5.2%, compared with 88.7% ± 3.1% for free DE and 92.9% ± 2.7% for ChNPs. At higher concentrations, UA-ChNPs/DE exhibited markedly enhanced cytotoxic activity, reducing cell viability to 40.0% ± 7.0% at 25 μg/mL and 25.2% ± 8.0% at 50 μg/mL. In comparison, free DE induced a more moderate reduction, reaching 49.4% ± 5.1% at 50 μg/mL, whereas ChNPs maintained high viability (>85%) across all concentrations, confirming the intrinsic biocompatibility of the carrier system. These findings demonstrate that nanoencapsulation significantly enhances the antiproliferative effect of dandelion extract.

**TABLE 1:** Dose-dependent cell viability % with IC_50_ of UA-ChNPs/DE, DE Extract alone and blank UA-ChNPs against HepG2 cells after 24 h of incubation.

​	Cell viability %			
Conc. (µg/mL)	UA-ChNPs/DE^*^	DE extract^*^	Blank UA-ChNPs (control)^*^	*P*-value
1.0	92.1 ± 3.4^a^	94.3 ± 2.9^a^	96.0 ± 2.1^a^	0.214
10	60.8 ± 5.2^b^	88.7 ± 3.1^a^	92.9 ± 2.7^a^	<0.001
25	40.0 ± 7.0^c^	75.2 ± 4.5^b^	88.0 ± 3.7^a^	<0.001
50	25.2 ± 8.0^c^	49.4 ± 5.1^b^	85.0 ± 2.2^a^	<0.001
IC_50_ (µg/mL)	17.8	49.4	>50	​

*Data are presented as mean ± SD (n = 3). *P*-values were calculated using one-way ANOVA, followed by Tukey’s multiple comparisons test. Different superscript letters (a, b, c) within the same row indicate statistically significant differences (*P* < 0.05).

Consistent with these findings, IC_50_ analysis demonstrated a markedly lower IC_50_ value for UA-ChNPs/DE (17.8 μg/mL) compared with free DE (49.4 μg/mL), corresponding to an approximately 2.8-fold enhancement in antiproliferative potency. Blank ChNPs exhibited negligible cytotoxicity (IC_50_ > 50 μg/mL), confirming that the observed effect is primarily attributable to the encapsulated extract.

#### 3.5.2 Comparative cytotoxicity across hepatocellular carcinoma cell lines

To evaluate the reproducibility of the anticancer effect, cytotoxicity was further assessed in Huh7 cells. UA-ChNPs/DE retained strong cytotoxic activity in Huh7 cells, with an IC_50_ value of 21.5 μg/mL, which was closely comparable to that observed in HepG2 cells (17.8 μg/mL). This consistency indicates that the enhanced cytotoxic effect of the nanoformulation is reproducible across different hepatocellular carcinoma models. In contrast, free DE showed moderate cytotoxicity in Huh7 cells (IC_50_ = 54.8 μg/mL), consistent with its lower potency in HepG2 cells. ChNPs did not exhibit significant cytotoxic effects in either cancer cell line (IC_50_ > 50 μg/mL), further confirming the biocompatibility and safety of the nanocarrier system.

#### 3.5.3 Cytotoxic selectivity in normal hepatic cells

The selectivity of the formulations was evaluated in LO2 normal hepatic cells under identical conditions (Table 2). UA-ChNPs/DE exhibited reduced cytotoxicity in LO2 cells, with an IC_50_ value of 52.4 μg/mL, which is substantially higher than those observed in the cancer cell lines.

**TABLE 2:** IC_50_ values and selectivity index (SI) of UA-ChNPs/DE, DE extract, and blank ChNPs across different cell lines.

Sample	HepG2 IC_50_ (µg/mL)^*^	Huh7 IC_50_ (µg/mL)^*^	LO2 IC_50_ (µg/mL)^*^	SI (HepG2)	SI (Huh7)
UA-ChNPs/DE	17.8	21.5	52.4	2.94	2.54
DE extract	49.4	54.8	68.2	1.38	1.25
Blank ChNPs	>50	>50	>50	—	—

*Data are expressed as IC_50_ values (µg/mL) derived from nonlinear regression analysis of dose–response curves following 24 h exposure.

This differential response indicates preferential cytotoxicity toward malignant cells. Free DE demonstrated weaker selectivity, with an IC_50_ value of 68.2 μg/mL in LO2 cells, indicating limited discrimination between normal and cancer cells. ChNPs remained minimally cytotoxic in LO2 cells (IC_50_ > 50 μg/mL), confirming their favorable biocompatibility.

#### 3.5.4 Selectivity index and integrated interpretation

Selectivity index (SI) analysis, calculated from IC_50_ values in cancer and normal cell lines (Table 2), further confirmed the enhanced therapeutic selectivity of UA-ChNPs/DE. SI values were 2.94 for HepG2 and 2.54 for Huh7 cells, indicating preferential cytotoxicity toward malignant cells. In contrast, free DE exhibited lower SI values (1.38 and 1.25), reflecting a narrower therapeutic window. Nanoencapsulation significantly improved both antiproliferative potency and selective toxicity toward cancer cells. The consistent IC_50_ values across hepatocellular carcinoma cell lines, together with the elevated SI values, highlight the superior therapeutic profile of UA-ChNPs/DE compared with free DE and underscore the functional advantage of the nanoformulation system.

### 3.6 Apoptosis induction assessed by Annexin V-FITC/PI flow cytometry

**FIGURE 5:**
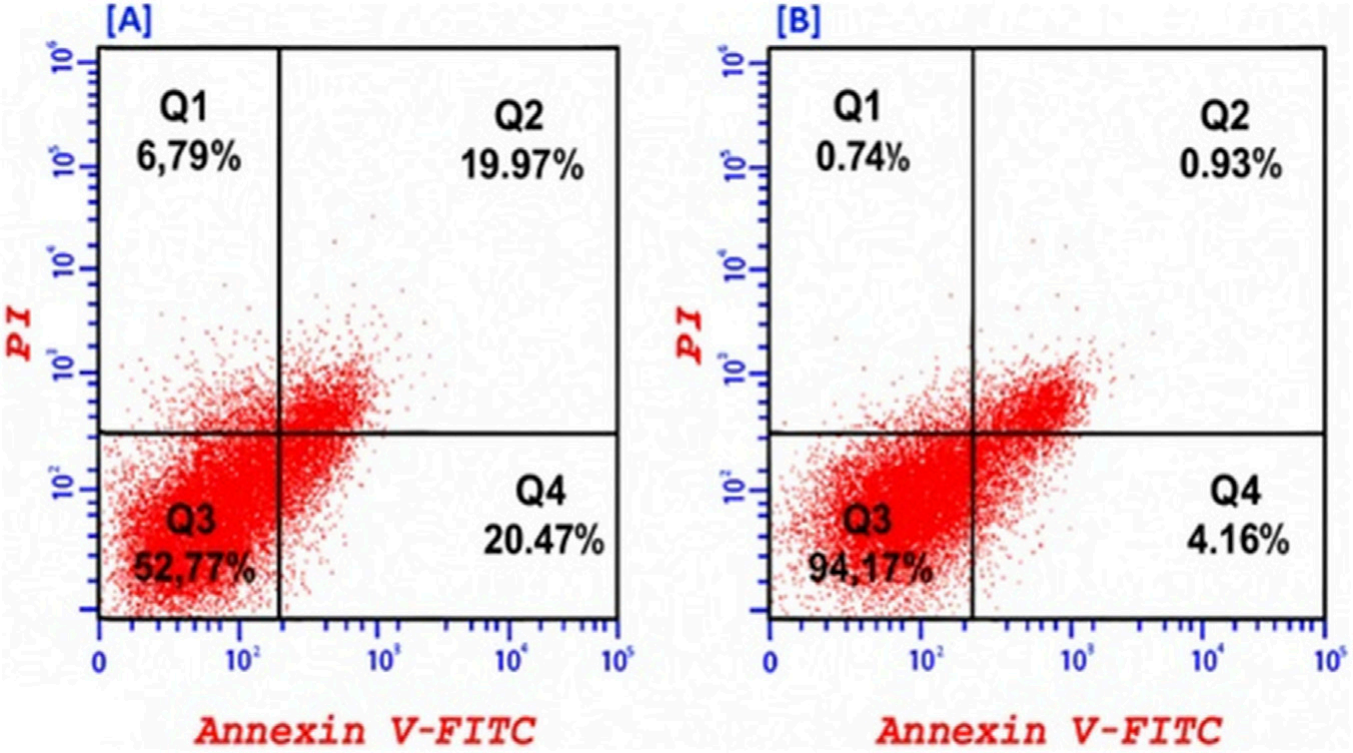
Flow cytometric analysis of apoptosis in HepG2 cells following treatment with UA-ChNPs/DE. HepG2 cells were treated with the IC_50_ concentration (17.8 μg/mL) of UA-ChNPs/DE **(A)** and compared with untreated control cells **(B)**. The quadrants represent Q1 (Annexin V^−^/PI^+^), necrotic cells; Q2 (Annexin V^+^/PI^+^), late apoptotic cells; Q3 (Annexin V^−^/PI^−^), viable cells; and Q4 (Annexin V^+^/PI^−^), early apoptotic cells.

The apoptotic effects of UA-ChNPs/DE in HepG2 hepatocellular carcinoma cells were quantitatively evaluated using Annexin V-FITC/propidium iodide (PI) dual staining followed by flow cytometry analysis. Representative dot plots of HepG2 cells treated with UA-ChNPs/DE at the IC_50_ concentration (17.8 μg/mL) and untreated control cells are presented in Figures 5A,B, respectively. In untreated HepG2 cells, most of the cell population was localized in the viable quadrant (Q3; Annexin V^−^/PI^−^), accounting for 94.17% of the total cells, reflecting minimal basal apoptosis. Only small fractions of early apoptotic (Q4; 4.16%), late apoptotic (Q2; 0.93%), and necrotic cells (Q1; 0.74%) were detected, confirming the physiological integrity of the control cell population. In contrast, treatment with UA-ChNPs/DE resulted in a pronounced redistribution of the cell population toward the apoptotic quadrants. The percentage of viable cells (Q3) decreased markedly to 52.77%, indicating a substantial loss of cell viability following treatment. This reduction was accompanied by a significant increase in early apoptotic cells (Q4; Annexin V^+^/PI^−^), which reached 20.47%, as well as a marked elevation in late apoptotic cells (Q2; Annexin V^+^/PI^+^), accounting for 19.97% of the total cell population. Additionally, a modest increase in necrotic cells (Q1; Annexin V^−^/PI^+^) was observed (6.79%), although this fraction remained comparatively low relative to the apoptotic populations.

The combined proportion of apoptotic cells (early and late apoptosis) increased from 5.09% in untreated controls to 40.44% following UA-ChNPs/DE treatment, demonstrating a strong pro-apoptotic effect. Importantly, the predominance of apoptotic rather than necrotic cell death indicates that UA-ChNPs/DE primarily trigger programmed cell death pathways rather than causing nonspecific cytotoxicity. These findings corroborate the MTT assay results and confirm that UA-ChNPs/DE induce programmed cell death in HepG2 cells.

## 4 Discussion

### 4.1 Formulation rationale and nanocarrier design

The present study demonstrates that ultrasound-assisted (UA) ionic gelation is an effective platform for engineering chitosan-based nanocarriers encapsulating dandelion extract with optimized physicochemical and release properties. The selection of chitosan as the polymeric matrix was strategically justified by its biocompatibility, biodegradability, low immunogenicity, and intrinsic cationic nature (Cheung et al., 2015). Chitosan contains protonatable amino groups that enable electrostatic interactions with polyanionic crosslinkers and negatively charged biological membranes, thereby facilitating nanoparticle formation and enhancing cellular uptake. These physicochemical characteristics have made chitosan one of the most widely investigated natural polymers for drug delivery and cancer nanomedicine (Stefanache et al., 2025). Moreover, chitosan has been reported to exhibit mild intrinsic anti-proliferative and antiangiogenic effects in HCC models, potentially providing additional therapeutic benefit without contributing significant cytotoxicity at formulation-relevant concentrations (Xu et al., 2009). Importantly, its ability to protect labile phytochemicals from premature degradation makes it particularly suitable for encapsulating bioactive plant constituents such as taraxasterol and taraxerol, the principal anticancer triterpenoids identified in dandelion (*Taraxacum officinale*) (Jiao et al., 2022). These phytochemicals have been reported to inhibit tumor cell proliferation, induce apoptosis, and modulate multiple signaling pathways associated with hepatocellular carcinoma progression (Ren et al., 2022; Herrera Vielma et al., 2025). Therefore, encapsulating dandelion extract within chitosan nanoparticles is expected to improve the stability, intracellular delivery, and therapeutic performance of these bioactive constituents.

### 4.2 Nanoscale engineering, crosslinking, and structural optimization

Nanoparticle-based delivery was adopted to address the inherent limitations of dandelion extract, including poor aqueous solubility, instability, and limited bioavailability (Desai et al., 2025). Reduction to the nanoscale (∼105 nm) significantly increases the surface-area-to-volume ratio, enhances dissolution behavior, and promotes cellular internalization through endocytic pathways (Ghazal et al., 2024). Furthermore, nanoparticles within the 50–200 nm size range are generally considered favorable for passive tumor accumulation through the enhanced permeability and retention (EPR) effect, although this phenomenon requires *in vivo* confirmation for each formulation (Venturini et al., 2025). The narrow size distribution (PDI < 0.3) obtained in this study suggests a homogeneous nanoparticle population with improved colloidal stability and more predictable biological interactions (Khoerunnisa et al., 2021).

The incorporation of sodium tripolyphosphate (TPP) as a crosslinking agent was essential for the formation of stable nanoparticles (Di Santo et al., 2021). Ionic gelation occurs through electrostatic interactions between positively charged chitosan amino groups and negatively charged phosphate groups of TPP, resulting in the spontaneous formation of a three-dimensional crosslinked network. Optimization of the TPP concentration (0.2% w/v) proved critical; insufficient crosslinking led to unstable and larger particles, whereas excessive TPP induced aggregation due to over-crosslinking and network densification. The selected TPP-to-chitosan ratio therefore ensured structural integrity, minimized aggregation, and contributed to the controlled-release behavior observed in the present study. UA processing further enhanced nanoparticle uniformity and dispersion stability. Acoustic cavitation generates localized high shear forces and microstreaming effects that promote efficient mixing, reduce polymer entanglement, and prevent premature aggregation during ionic crosslinking (Herdiana, 2025). However, excessive amplitude increased particle size, indicating that optimal cavitation intensity is required to balance size reduction against potential re-agglomeration. The optimized conditions (40% amplitude, 750 W, pulsed mode) enabled the production of spherical, discrete nanoparticles, as confirmed by SEM analysis. The preservation of particle morphology without structural collapse underscores the advantage of ultrasound-assisted processing in producing homogeneous nanocarriers without the need for harsh chemical conditions or elevated temperatures that could degrade thermolabile phytochemicals (Wu et al., 2022). This interpretation is further supported by the uniform particle-size distribution and positive zeta potential obtained in the present study, both of which indicate successful formulation optimization and favorable colloidal stability (Khoerunnisa et al., 2021; Ghazal et al., 2024).

### 4.3 Solid-state behavior and release modulation

XRPD analysis revealed a marked attenuation of the crystalline peaks of the dandelion extract following encapsulation, indicating a partial transition toward an amorphous state within the chitosan matrix. Amorphization enhances apparent solubility and dissolution kinetics by increasing molecular dispersion and reducing lattice-energy constraints (Podgorbunskikh et al., 2022). Such structural modification is particularly relevant for hydrophobic triterpenoids such as taraxasterol and taraxerol, whose therapeutic potential is often limited by aqueous solubility and low bioavailability (Yan et al., 2024). The disappearance or reduction of extract-specific diffraction peaks suggests the molecular-level incorporation of the phytochemicals within the polymeric network rather than their superficial adsorption, thereby supporting the successful encapsulation of the bioactive constituents (Di Santo et al., 2021).

The *in vitro* release profile further highlights the functional significance of nanoencapsulation in modulating the release behavior of dandelion phytoconstituents. Free dandelion extract demonstrated rapid diffusion in PBS, approaching near-complete release within 72 h, which is characteristic of unencapsulated bioactive compounds that readily diffuse in aqueous media (Di Santo et al., 2021). In contrast, UA-ChNPs/DE exhibited a controlled biphasic release profile, with an initial release of approximately 12% during the first hour, followed by a gradual and sustained increase, reaching nearly 71% at 72 h. The moderate initial release phase likely corresponds to the release of surface-associated or loosely entrapped molecules, whereas the subsequent prolonged release phase is primarily governed by diffusion through the ionically crosslinked chitosan–TPP matrix. The electrostatic interactions between protonated amino groups of chitosan and TPP create a compact polymeric network that restricts rapid molecular transport, thereby attenuating the initial burst release and extending drug release over time (Naskar et al., 2019).

The diffusion-controlled nature of the release process was further supported by the strong fit obtained with the Higuchi model. This observation is consistent with previous reports describing sustained release from chitosan-based nanoparticle systems, where diffusion through the polymeric matrix represents the predominant release mechanism (Petropoulos et al., 2012; Naskar et al., 2019). Similar sustained-release behavior has also been reported for ultrasound-fabricated polymeric nanocarriers, where controlled diffusion contributes to prolonged therapeutic exposure and improved formulation performance (Wu et al., 2022). This sustained delivery profile is particularly advantageous for anticancer applications because it can maintain prolonged cellular exposure to active phytochemicals while reducing rapid concentration fluctuations associated with free extract administration. The modified release kinetics of UA-ChNPs/DE provide mechanistic support for the enhanced cytotoxic efficacy observed *in vitro* and demonstrate the ability of the nanoparticle system to overcome key pharmacokinetic limitations of unencapsulated plant-derived compounds (Ammar et al., 2025).

### 4.4 Cytotoxicity enhancement and mechanistic anticancer effects

The biological findings of the present study clearly demonstrate that nanoencapsulation of dandelion extract within UA-ChNPs markedly enhances its antiproliferative efficacy against hepatocellular carcinoma cells. The MTT assay demonstrated that the IC_50_ value in HepG2 cells decreased significantly from 49.4 μg/mL for free DE to 17.8 μg/mL for UA-ChNPs/DE, representing an approximately 2.8-fold enhancement in cytotoxic potency. Importantly, blank ChNPs exhibited negligible cytotoxicity (IC_50_ > 50 μg/mL), confirming that the observed effect is primarily attributable to enhanced phytochemical delivery rather than carrier-induced cytotoxicity. A similar trend was observed in Huh7 cells, where UA-ChNPs/DE retained strong cytotoxic activity (IC_50_ = 21.5 μg/mL), demonstrating reproducible anticancer activity across distinct HCC phenotypes. In contrast, free DE remained less potent (IC_50_ = 54.8 μg/mL), whereas blank ChNPs exhibited minimal biological activity. In normal LO2 hepatic cells, UA-ChNPs/DE exhibited reduced cytotoxicity (IC_50_ = 52.4 μg/mL), indicating preferential toxicity toward malignant cells. This observation was further supported by the selectivity index values (2.94 for HepG2 and 2.54 for Huh7), which were substantially higher than those of free DE (1.38 and 1.25), confirming that nanoencapsulation broadened the therapeutic window while improving anticancer selectivity. The improved efficacy can be mechanistically interpreted through several complementary factors. The positive surface charge of chitosan nanoparticles promotes electrostatic interaction with negatively charged phospholipid membranes, enhancing cellular adhesion and uptake efficiency (Aibani et al., 2021). In addition, the sustained-release behavior of the crosslinked chitosan–TPP matrix likely maintains prolonged intracellular exposure to bioactive phytochemicals, thereby amplifying cytotoxic signaling pathways and improving the overall pharmacodynamic response (Ammar et al., 2025).

Dandelion extract contains bioactive pentacyclic triterpenoids, particularly taraxasterol and taraxerol, which have been reported to exert significant anticancer activity in HCC models (Yan et al., 2024). Taraxasterol has been shown to inhibit STAT3/IL-6 signaling, suppress proliferative markers such as Ki-67, and modulate immune-associated pathways involved in tumor progression (Jiao et al., 2022). Furthermore, recent studies have demonstrated that taraxasterol promotes apoptosis and suppresses hepatocellular carcinoma progression through the modulation of multiple oncogenic signaling pathways (Ren et al., 2022; Herrera Vielma et al., 2025). Similarly, taraxerol has demonstrated antiproliferative and pro-apoptotic activities through the regulation of mitochondrial integrity, caspase activation, and Bcl-2 family proteins (Huo et al., 2022). However, both compounds are relatively hydrophobic and exhibit limited aqueous solubility, thereby restricting their intracellular bioavailability in the free form. Encapsulation within chitosan nanoparticles likely improves their dispersion, cellular transport, and protection from premature degradation, thereby enhancing their effective intracellular concentration and pharmacodynamic activity (Di Santo et al., 2021). These mechanisms provide a plausible explanation for the enhanced cytotoxicity and selectivity observed for UA-ChNPs/DE in the present study and are consistent with the well-established intracellular delivery characteristics of chitosan-based nanocarriers (Ghazal et al., 2024; Stefanache et al., 2025).

### 4.5 Apoptosis induction and cell death mechanisms

The apoptosis analysis further substantiates the enhanced anticancer activity of UA-ChNPs/DE. Treatment at the IC_50_ concentration resulted in a marked increase in the total apoptotic cell population (∼40%) compared with untreated controls (∼5%), with significant increases in both early and late apoptotic cell populations. Importantly, apoptosis was the predominant mode of cell death, indicating activation of regulated cell death pathways rather than nonspecific membrane disruption. This distinction is therapeutically relevant because apoptosis-based anticancer strategies are generally associated with reduced inflammatory sequelae and improved tolerability compared with necrotic cell death (Pistritto et al., 2016). Therefore, the predominance of apoptotic over necrotic cell death observed in the present study suggests that UA-ChNPs/DE induce a controlled cytotoxic response while potentially minimizing off-target inflammatory effects.

The enhanced apoptotic response is likely attributable to improved intracellular delivery and sustained exposure of HCC cells to bioactive phytochemicals following nanoencapsulation. Taraxasterol and related triterpenoids have previously been reported to induce apoptosis through the modulation of mitochondrial signaling, activation of caspase cascades, and suppression of prosurvival pathways, including STAT3/IL-6 signaling (Ren et al., 2022; Herrera Vielma et al., 2025). Although these molecular pathways were not directly investigated in the present study, the substantial increase in apoptotic cells is consistent with these previously reported mechanisms and supports the hypothesis that nanoencapsulation enhances the biological activity of dandelion-derived phytochemicals. Future mechanistic studies evaluating caspase activation, mitochondrial membrane potential, oxidative stress biomarkers, and apoptosis-related signaling pathways will further clarify the molecular basis of the observed pro-apoptotic effects.

### 4.6 Translational relevance and therapeutic implications

From a translational perspective, the integration of phytochemical-based therapy with nanotechnology offers a rational strategy to address persistent challenges in HCC management. HCC continues to be associated with high recurrence rates, therapeutic resistance, and limited responsiveness to conventional chemotherapy (Kumari et al., 2018; Mauro et al., 2025). Natural compounds such as taraxasterol and taraxerol exhibit promising anticancer properties but have historically faced barriers related to solubility, bioavailability, and pharmacokinetic instability (Andreani et al., 2024). The UA-ChNPs/DE nanoformulation developed in the present study was specifically designed to overcome these limitations by combining chitosan-mediated protection, TPP-stabilized ionic crosslinking, and ultrasound-assisted nanoscale engineering into a structurally stable and biologically effective delivery platform. The enhanced cytotoxic activity observed across multiple HCC cell lines, together with improved selectivity toward normal hepatic cells, demonstrates the potential of UA-ChNPs/DE to improve the therapeutic index of dandelion-derived phytochemicals. Furthermore, the sustained-release characteristics and favorable physicochemical properties of the nanoformulation may contribute to prolonged drug exposure, enhanced intracellular delivery, and improved therapeutic performance compared with the free extract (Ghazal et al., 2024; Ammar et al., 2025). The findings also suggest a potential synergistic interaction between the intrinsic biological properties of chitosan and the encapsulated phytochemicals. Although blank chitosan nanoparticles exhibited minimal cytotoxicity, the cationic nature, mucoadhesive characteristics, and permeability-enhancing properties of chitosan are known to facilitate cellular interaction, promote intracellular uptake, and improve the retention of encapsulated therapeutic agents (Jiménez-Gómez and Cecilia, 2020). These physicochemical advantages likely contributed to the enhanced antiproliferative activity observed in the present study.

The present findings are also consistent with previous reports demonstrating that chitosan-based nanocarriers enhance the therapeutic efficacy of plant-derived anticancer compounds by improving formulation stability, cellular internalization, and controlled-release behavior (Di Santo et al., 2021; Ghazal et al., 2024). Moreover, ultrasound-assisted preparation further strengthens the translational potential of this delivery platform by providing a simple, scalable, reproducible, and relatively mild fabrication method that avoids harsh organic solvents or elevated processing temperatures that could compromise phytochemical integrity (Wu et al., 2022).

### 4.7 Challenges, future perspectives, and study limitations

The present findings demonstrate that ultrasound-assisted chitosan nanoencapsulation represents a promising strategy for improving the therapeutic performance of dandelion-derived phytochemicals against hepatocellular carcinoma. Future research should focus on advancing this nanoformulation toward preclinical and translational applications through comprehensive pharmacokinetic, pharmacodynamic, efficacy, and safety evaluations in appropriate animal models. In addition, mechanistic investigations involving apoptosis signaling, oxidative stress modulation, inflammatory pathways, cellular uptake, and intracellular trafficking will provide a deeper understanding of the molecular mechanisms responsible for the enhanced anticancer activity. Furthermore, optimization of the formulation for large-scale production, long-term stability, and regulatory compliance will be essential to facilitate future clinical translation.

Despite these promising prospects, several challenges remain. One of the major challenges associated with plant-derived nanoformulations is the inherent variability in phytochemical composition among different extract batches, which may influence formulation reproducibility and therapeutic consistency. In addition, maintaining nanoparticle stability during long-term storage, achieving scalable and cost-effective manufacturing, and ensuring batch-to-batch reproducibility represent important challenges that should be addressed before clinical implementation. Another challenge is the complex biological behavior of nanoparticle systems *in vivo*, including biodistribution, protein corona formation, immune interactions, and potential off-target accumulation, all of which require comprehensive investigation.

Several study limitations should also be acknowledged. First, although the present study demonstrated consistent anticancer activity in two hepatocellular carcinoma cell lines (HepG2 and Huh7), further validation in additional HCC models, particularly drug-resistant and patient-derived models, would strengthen the generalizability of the findings. Second, although apoptosis induction was confirmed by flow cytometry, detailed molecular investigations, including caspase activation, mitochondrial membrane potential analysis, STAT3/IL-6 signaling, NF-κB modulation, oxidative stress biomarkers, cell-cycle regulation, and apoptosis-related gene and protein expression, were beyond the scope of the present study and should be incorporated in future investigations to elucidate the underlying molecular mechanisms. Third, *in vivo* pharmacokinetic profiling, biodistribution, systemic toxicity, histopathological evaluation, and antitumor efficacy studies were not performed and remain essential for confirming the translational potential of UA-ChNPs/DE under physiological conditions. Accordingly, a dedicated follow-up study is currently underway to investigate these aspects in experimental hepatocellular carcinoma animal models.

The *in vitro* release study was conducted under physiological conditions (PBS, pH 7.4) to evaluate formulation stability and release behavior. Future studies should additionally investigate release under mildly acidic tumor-mimicking conditions (pH 6.5–6.8) to better simulate the tumor microenvironment. Moreover, release quantification was based on the measured taraxasterol content, which reflects the overall release behavior of the dandelion extract rather than the independent release kinetics of individual phytochemicals such as taraxasterol and taraxerol. Future studies employing validated chromatographic profiling after nanoparticle disruption will enable compound-specific release characterization and clarify the relative contribution of individual phytoconstituents to the observed anticancer activity.

Nevertheless, these challenges and limitations do not diminish the principal findings of the present study. Rather, they define a clear roadmap for future mechanistic, pharmacological, and translational investigations aimed at advancing ultrasound-assisted chitosan nanoparticle delivery of dandelion-derived phytochemicals toward preclinical development and, ultimately, clinical application for hepatocellular carcinoma therapy.

## 5 Conclusion

The present study demonstrates that ultrasound-assisted chitosan nanoparticle engineering represents an effective strategy for enhancing the therapeutic potential of dandelion (*Taraxacum officinale*) extract against hepatocellular carcinoma. The optimized UA-ChNPs/DE nanoformulation exhibited favorable physicochemical characteristics, including nanoscale particle size, narrow size distribution, positive surface charge, partial amorphization of the encapsulated phytochemicals, and sustained-release behavior, all of which contributed to improved formulation performance. Nanoencapsulation significantly enhanced the antiproliferative activity of dandelion extract, increased apoptotic cell death, and improved selective cytotoxicity toward hepatocellular carcinoma cells while reducing toxicity toward normal hepatic cells. Collectively, these findings demonstrate that ultrasound-assisted chitosan nanoencapsulation effectively overcomes key physicochemical and delivery limitations associated with plant-derived bioactive compounds and represents a promising nanotherapeutic platform for hepatocellular carcinoma treatment. Future investigations should focus on comprehensive molecular mechanism studies together with *in vivo* pharmacokinetic, biodistribution, safety, and therapeutic efficacy evaluations to facilitate the preclinical and clinical translation of this phytochemical-based nanomedicine.

## Data Availability

The original contributions presented in the study are included in the article/supplementary material, further inquiries can be directed to the corresponding author.

## References

[B1] Aibani N. Rai R. Patel P. Cuddihy G. Wasan E. K. (2021). Chitosan nanoparticles at the biological interface: implications for drug delivery. Pharmaceutics 13, 1686. 10.3390/pharmaceutics13101686 34683979 PMC8540112

[B2] Ammar M. M. Ali R. Abd Elaziz N. A. Habib H. Abbas F. M. Yassin M. T. (2025). Nanotechnology in oncology: advances in biosynthesis, drug delivery, and theranostics. Discov. Oncol. 16, 1172. 10.1007/s12672-025-02664-3 40542991 PMC12182552

[B3] Andreani T. Cheng R. Elbadri K. Ferro C. Menezes T. dos Santos M. R. (2024). Natural compounds-based nanomedicines for cancer treatment: future directions and challenges. Drug Deliv. Transl. Res. 14, 2845–2916. 10.1007/s13346-024-01649-z 39003425 PMC11385056

[B4] Cheung R. Ng T. Wong J. Chan W. (2015). Chitosan: an update on potential biomedical and pharmaceutical applications. Mar. Drugs 13, 5156–5186. 10.3390/md13085156 26287217 PMC4557018

[B5] Cui D. Zhang C. Zhang L. Zheng J. Wang J. He L. (2025). Natural anti-cancer products: insights from herbal medicine. Chin. Med. 20, 82. 10.1186/s13020-025-01124-y 40490812 PMC12147394

[B6] Dalzell C. G. Taylor A. C. White S. B. (2023). New insights on liver-directed therapies in hepatocellular carcinoma. Cancers (Basel) 15, 5749. 10.3390/cancers15245749 38136295 PMC10741466

[B7] Desai N. Rana D. Patel M. Bajwa N. Prasad R. Vora L. K. (2025). Nanoparticle therapeutics in clinical perspective: classification, marketed products, and regulatory landscape. Small 21, e2502315. 10.1002/smll.202502315 40454890 PMC12288819

[B8] Di Santo M. C. D’Antoni C. L. Domínguez Rubio A. P. Alaimo A. Pérez O. E. (2021). Chitosan-tripolyphosphate nanoparticles designed to encapsulate polyphenolic compounds for biomedical and pharmaceutical applications − A review. Biomed. Pharmacother. 142, 111970. 10.1016/j.biopha.2021.111970 34333289

[B9] Ghazal H. Waqar A. Yaseen F. Shahid M. Sultana M. Tariq M. (2024). Role of nanoparticles in enhancing chemotherapy efficacy for cancer treatment. Next Mater. 2, 100128. 10.1016/j.nxmate.2024.100128

[B10] Herdiana Y. (2025). Nanoparticles of natural product-derived medicines: beyond the pandemic. Heliyon 11, e42739. 10.1016/j.heliyon.2025.e42739 40083991 PMC11904502

[B11] Herrera Vielma F. Quiñones San Martin M. Muñoz-Carrasco N. Berrocal-Navarrete F. González D. R. Zúñiga-Hernández J. (2025). The role of dandelion (*Taraxacum officinale*) in liver health and hepatoprotective properties. Pharmaceuticals 18, 990. 10.3390/ph18070990 40732279 PMC12299503

[B33] Huo B. Song Y. Tan B. Li J. Zhang J. Zhang F. (2022). Research on the mechanisms of taraxerol for the treatment of gastric cancer effect based on network pharmacology. Int. J. Immunopathol. Pharmacol. 36. 10.1177/20587384211063962 PMC8743941 34986036

[B12] Jiao F. Tan Z. Yu Z. Zhou B. Meng L. Shi X. (2022). The phytochemical and pharmacological profile of taraxasterol. Front. Pharmacol. 13, 927365. 10.3389/fphar.2022.927365 35991893 PMC9386448

[B13] Jiménez-Gómez C. P. Cecilia J. A. (2020). Chitosan: a natural biopolymer with a wide and varied range of applications. Molecules 25 (17), 3981. 10.3390/molecules25173981 32882899 PMC7504732

[B14] Khoerunnisa F. Yolanda Y. D. Nurhayati M. Zahra F. Nasir M. Opaprakasit P. (2021). Ultrasonic synthesis of nanochitosan and its size effects on turbidity removal and dealkalization in wastewater treatment. Inventions 6, 98. 10.3390/inventions6040098

[B15] Krupa K. Fudalej M. Cencelewicz-Lesikow A. Badowska-Kozakiewicz A. Czerw A. Deptała A. (2024). Current treatment methods in hepatocellular carcinoma. Cancers (Basel) 16, 4059. 10.3390/cancers16234059 39682245 PMC11640602

[B16] Kumari R. Sahu M. K. Tripathy A. Uthansingh K. Behera M. (2018). Hepatocellular carcinoma treatment: hurdles, advances and prospects. Hepat. Oncol. 5, HEP08. 10.2217/hep-2018-0002 31293776 PMC6613045

[B17] Mauro E. de Castro T. Zeitlhoefler M. Sung M. W. Villanueva A. Mazzaferro V. (2025). Hepatocellular carcinoma: epidemiology, diagnosis and treatment. JHEP Rep. 7, 101571. 10.1016/j.jhepr.2025.101571 41244300 PMC12615749

[B34] Mosmann T. (1983). Rapid colorimetric assay for cellular growth and survival: application to proliferation and cytotoxicity assays. J. Immunol. Methods. 65 (1), 55–63. 10.1016/0022-1759(83)90303-4 6606682

[B18] Naskar S. Sharma S. Kuotsu K. (2019). Chitosan-based nanoparticles: an overview of biomedical applications and its preparation. J. Drug Deliv. Sci. Technol. 49, 66–81. 10.1016/J.JDDST.2018.10.022

[B19] Petropoulos J. H. Papadokostaki K. G. Sanopoulou M. (2012). Higuchi’s equation and beyond: overview of the formulation and application of a generalized model of drug release from polymeric matrices. Int. J. Pharm. 437, 178–191. 10.1016/J.IJPHARM.2012.08.012 22921377

[B20] Pistritto G. Trisciuoglio D. Ceci C. Garufi A. D’Orazi G. (2016). Apoptosis as anticancer mechanism: function and dysfunction of its modulators and targeted therapeutic strategies. Aging 8, 603–619. 10.18632/aging.100934 27019364 PMC4925817

[B21] Podgorbunskikh E. Kuskov T. Rychkov D. Lomovskii O. Bychkov A. (2022). Mechanical amorphization of chitosan with different molecular weights. Polym. (Basel) 14, 4438. 10.3390/polym14204438 PMC9606905 36298017

[B22] Ren F. Zhang Y. Qin Y. Shang J. Wang Y. Wei P. (2022). Taraxasterol prompted the anti-tumor effect in mice burden hepatocellular carcinoma by regulating T lymphocytes. Cell Death Discov. 8, 264. 10.1038/s41420-022-01059-5 35577774 PMC9110731

[B23] Rîmbu M. C. Cord D. Savin M. Grigoroiu A. Mihăilă M. A. Gălățanu M. L. (2025). Harnessing plant-based nanoparticles for targeted therapy: a green approach to cancer and bacterial infections. Int. J. Mol. Sci. 26, 7022. 10.3390/ijms26147022 40725269 PMC12296188

[B24] Sharma K. Zafar R. (2014). Simultaneous estimation of taraxerol and taraxasterol in root callus cultures of *Taraxacum officinale* Weber. Int. J. Pharmacogn. Phytochem. Res. 6, 540–546.

[B25] Shittu R. O. Ceesay I. Pwavodi P. C. (2025). Antioxidant and antimicrobial activities of Dandelion root extract (*Taraxacum officinale*) and its cytotoxic effect on MDA-MB-231 breast cancer cells. Discov. Appl. Sci. 7, 136. 10.1007/s42452-024-06419-7

[B26] Situmorang P. C. Ilyas S. Nugraha S. E. Syahputra R. A. Nik Abd Rahman N. M. A. (2024). Prospects of compounds of herbal plants as anticancer agents: a comprehensive review from molecular pathways. Front. Pharmacol. 15, 1387866. 10.3389/fphar.2024.1387866 39104398 PMC11298448

[B27] Stefanache A. Lungu I. I. Anton N. Damir D. Gutu C. Olaru I. (2025). Chitosan nanoparticle-based drug delivery systems: advances, challenges, and future perspectives. Polym. (Basel) 17, 1453. 10.3390/polym17111453 PMC12157975 40508696

[B28] Venturini J. Chakraborty A. Baysal M. A. Tsimberidou A. M. (2025). Developments in nanotechnology approaches for the treatment of solid tumors. Exp. Hematol. Oncol. 14, 76. 10.1186/s40164-025-00656-1 40390104 PMC12090476

[B29] Wang A. Xiong W. Cheng C. Zou L. Niu B. Liu Y. (2025). Tracking evidences of dandelion for the treatment of cancer: from chemical composition, bioactivity, signaling pathways in cancer cells to perspective study. Nutrients 17, 3769. 10.3390/nu17233769 41374059 PMC12694484

[B30] Wu Z. Li Y. Tang J. Lin D. Qin W. Loy D. A. (2022). Ultrasound-assisted preparation of chitosan/nano-silica aerogel/tea polyphenol biodegradable films: physical and functional properties. Ultrason. Sonochem. 87, 106052. 10.1016/j.ultsonch.2022.106052 35660275 PMC9168617

[B31] Xu Y. Wen Z. Xu Z. (2009). Chitosan nanoparticles inhibit the growth of human hepatocellular carcinoma xenografts through an antiangiogenic mechanism. Anticancer Res. 29, 5103–5109. 20044623

[B32] Yan Q. Xing Q. Liu Z. Zou Y. Liu X. Xia H. (2024). The phytochemical and pharmacological profile of dandelion. Biomed. Pharmacother. 179, 117334. 10.1016/j.biopha.2024.117334 39180794

